# Large-Scale Green Liver System for Sustainable Purification of Aquacultural Wastewater: Construction and Case Study in a Semiarid Area of Brazil (Itacuruba, Pernambuco) Using the Naturally Occurring Cyanotoxin Microcystin as Efficiency Indicator

**DOI:** 10.3390/toxins12110688

**Published:** 2020-10-30

**Authors:** Maranda Esterhuizen, Stephan Pflugmacher

**Affiliations:** 1 Ecosystems and Environmental Research Programme, Faculty of Biological and Environmental Sciences, University of Helsinki, Niemenkatu 73, 15140 Lahti, Finland; Stephan.PflugmacherLima@umanitoba.ca; 2 Helsinki Institute of Sustainability Science (HELSUS), University of Helsinki, Fabianinkatu 33, 00014 Helsinki, Finland; 3 Korea Institute of Science and Technology Europe (KIST), Joint Laboratory of Applied Ecotoxicology, Campus 7.1, 66123 Saarbrücken, Germany; 4 Clayton H. Riddell Faculty of Environment, Earth, and Resources, University of Manitoba, Wallace Bldg, 125 Dysart Rd, Winnipeg, MB R3T 2N2, Canada

**Keywords:** phytoremediation, cyanobacterial toxins, microcystin degradation, water treatment, ecosystem services

## Abstract

The aquaculture industry in Brazil has grown immensely resulting in the production of inefficiently discarded wastewater, which causes adverse effects on the aquatic ecosystem. The efficient treatment of aquaculture wastewater is vital in reaching a sustainable and ecological way of fish farming. Bioremediation in the form of the Green Liver System employing macrophytes was considered as wastewater treatment for a tilapia farm, COOPVALE, in Itacuruba, Brazil, based on previously demonstrated success. A large-scale system was constructed, and the macrophytes *Azolla caroliniana*, *Egeria densa*, *Myriophyllum aquaticum*, and *Eichhornia crassipes* were selected for phytoremediation. As cyanobacterial blooms persisted in the eutrophic wastewater, two microcystin congeners (MC-LR and -RR) were used as indicator contaminants for system efficiency and monitored by liquid-chromatography–tandem-mass-spectrometry. Two trial studies were conducted to decide on the final macrophyte selection and layout of the Green Liver System. In the first trial, 58% MC-LR and 66% MC-RR were removed and up to 32% MC-LR and 100% MC-RR were removed in the second trial. Additional risks that were overcome included animals grazing on the macrophytes and tilapia were spilling over from the hatchery. The implementation of the Green Liver System significantly contributed to the bioremediation of contaminants from the fish farm.

## 1. Introduction

Brazil, in particular the Pantanal and the Amazon areas, is well known for its majestic landscapes, which are linked to vast water resources. Brazil holds approximately 10% of the global freshwater water supply stored in nearly 30,000 reservoirs covering a surface area of approximately 50,000 km^2^. Most of these reservoirs have been built for energy production, irrigation, and drought mitigation purposes. These seemingly infinite water sources have attracted a large variety of commercial fish farming. Most of them are small-scale production units; however, they summarize to about 100,000 aquacultural units occupying approximately 80,000 ha [1]. Besides fish, other aquatic organisms, such as shrimp (mostly *Litopenaeus vannamei*), crayfish (*Procambarus clarkii*), bivalves (*Mytilidae* and *Oyster*), as well as the bullfrog (*Rana catesbeiana*) are reared on a commercial scale. The fish species typically used are native to, e.g., the Parana, Sao Francisco, and the Amazon; however, Brazil has a long-term history for introducing alien fish species for aquacultural purposes as well. For example, the Nile tilapia (*Oreochromis niloticus*), introduced to Brazil in the 1950s, has become one of the most important fish species used commercially [2]. With the introduction of sex-reversal technology using hormones, the small volume/high-density cage technology (SVHD), rich nutrient food, and fish antibiotics, the farming of Nile tilapia has become more economically feasible with production yields reaching approximately 133,000 metric tons by 2009 [1].

**Figure 1:**
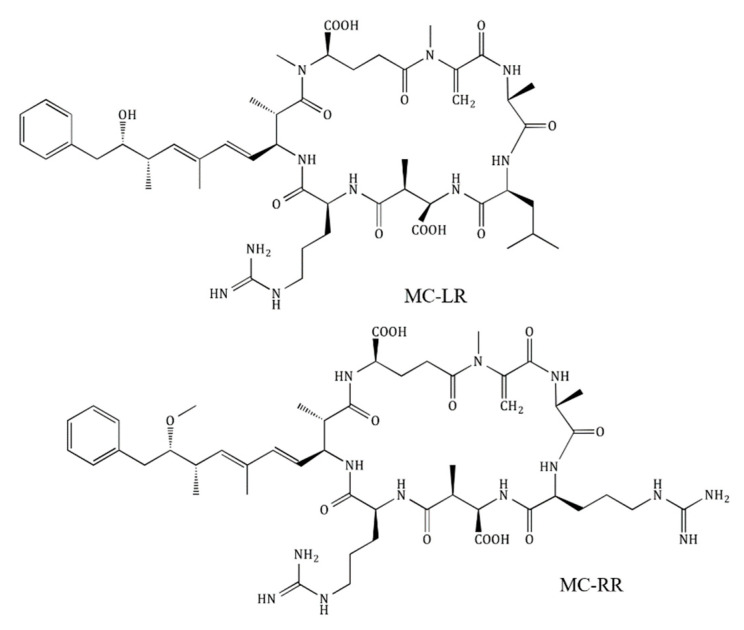
Chemical structure of the two microcystin congeners microcystin (MC)-LR and -RR.

Aquaculture enterprises typically employ land-based pond systems for fish hatching and rearing [3], resulting in wastewaters released into nearby reservoirs. The wastewaters would not only include hormones and antibiotics commonly used in aquaculture [4,5] but also would be enriched with nutrients leading to the eutrophication of these waterbodies and subsequently, the domination of cyanobacterial blooms [6]. Cyanobacteria do not only cause aesthetic issues in surface water but also produce a range of toxins, the most commonly detected being the microcystins (MCs) [7]. The hepatotoxic MCs can occur in various isoforms, differentiated only by variations of two amino acids in fixed positions; e.g., MC-LR contains lysine and arginine, and MC-RR contains two arginine residues (Figure 1).

In semiarid and arid regions of Brazil, the water from these reservoirs is not only used for irrigation, but also as a drinking water resource. The water quality of these reservoirs, therefore directly affects human health in the region. Due to the still-growing aquacultural industry in Brazil, along with the known associated environmental impacts [8]; it has become increasingly necessary to address the development of technologies that will ensure the sustained high quality of freshwater reservoirs in the wake of the aquaculture boom and ensure successful implementation.

Acknowledging freshwater as the limited resource that it is, it becomes apparent that tools are needed to purify water sustainably and cost-effectively to make it affordable for small unit enterprises. One option is the use of the Green Liver System^®^, which in contrast to the wetland system, is an entirely artificial system that utilizes the phytoremediation potential of aquatic plants, which take up and biotransform contaminants from water [9,10]. Plants and animals share many similarities regarding the biotransformation of xenobiotics, except in stage three, where animals secrete the biotransformed products and plants sequester them in cell wall fractions, the apoplast, or vacuoles [11,12]. Phytoremediation has shown promising results with respect to eutrophication, xenobiotics, and cyanobacterial toxin removal capabilities in the laboratory [10,13,14,15,16,17,18] as well as in a small pilot plant in Hefei (Anhui region, China) [19].

As with all water treatment options, several risks factors need to be considered before the Green Liver Systems can be implemented and continued sustainable operation assured. First, the main contaminants concerned have to be quantified to establish their concentrations in the wastewater. Here, the first step towards a customized Green Liver System takes place and plants are chosen according to the specific contaminants present. The selection of the individual species and combination of macrophytes is based on extensive laboratory research, which has established their remediation capabilities for the relevant contaminants and to avoid allelopathic effects between the different plant species [9].

After setting up the system, three major risk groups can disturb the running system; climate, nature, and humans. For the climate, factors such as heavy rain or drought might be a risk for the Green Liver System. Heavy rain can flush the system, causing the macrophytes to accumulate near the discharge points, causing them to die-off. Drought could cause decreasing water levels in combination with increasing water temperature leading to the death of the macrophytes as well. Besides climatic factors, animals, typically such as donkeys and wild goats in Brazil, could invade the system and feed on the fresh aquatic plants. Furthermore, as this system is designed to treat the wastewater of a tilapia farm, it is highly likely that juvenile fish might enter the system via the inflow. Tilapia fish are plant feeders and might significantly reduce the biomass of the aquatic macrophytes in the system, which would lead to a decrease in the efficiency of the system itself.

The present study aimed to construct a Green Liver System suitable for the treatment of wastewater from COOPVALE, a tilapia farm opened in 1999. Before the system had been constructed, water from the tilapia farm was directly released into the nearby Itaparica reservoir (now known as the Luiz Gonzaga Dam). The presented research tested the upscaling of a lab-scale [10] and small pilot-scale system [19] to a large-scale system in terms of flow rate and a retention time of three days to yield satisfactory water remediation. Due to the eutrophication resulting from the fish farm waste, cyanobacterial blooms occurred in the wastewater from COOPVALE. The two most commonly detected MC congeners, MC-LR and -RR, were selected as system efficiency indicator contaminants and were monitored at the fish farm hatchery, as well as the system inlet and outlet during two trials to investigate the remediation efficiency of the Green Liver System. The risks associated with the implementation of such a system was evaluated, and mitigation attempts were tested in the two trial studies.

## 2. Results and Discussion

The immense and still growing aquacultural industry in Brazil has created the need for sustainable wastewater purification to remove nutrients, toxins, and veterinary substances (such as hormones and antibiotics). The excessive application of fish food ad libitum caused the reservoir, as a sink for the wastewater, to become eutrophic [3] causing cyanobacterial blooms [6]. As the financial means of the Brazilian fish farmers in most cases are limited, it is necessary to use low-cost strategies to suit the financial situation of the farmers. Thus, treating aquacultural wastewater using aquatic plants, in particular, using the Green Liver System and employing native macrophytes, seems like the most feasible approach [9,20].

### 2.1. First Trial

Shortly after construction and planting, during the four-week macrophyte acclimation period, the large-scale Green Liver System was invaded by wild, formerly domestic, goats, which fed on the aquatic macrophytes. A goat-proof fence was constructed around the system to keep them out.

The aquatic fern *Azolla caroliniana*, known for its remediation of heavy metals [21], nitrate from water [22,23], and effluents from fish farms [23,24], was selected for the first compartment. In laboratory studies, *A. caroliniana* remediated up to 41% phosphate and 30% nitrate [24]. In the Green Liver System^®^, this free-floating fern was used to cover the water surface of the first compartment to limit sunlight penetration and thus the development of cyanobacterial blooms. The sunlight intensity in Brazil during summer (October to February), however, was too high and did not support the growth of *A. caroliniana*. After three weeks, the compartments housing *A. caroliniana* turned red and started dying. *A. caroliniana* requires low light intensities for growth, needing only 25–50% of sunlight (approximately 3200 lux) [25,26]. Optimal water temperature for the growth is 30 °C, whereas the growth rate was reduced significantly by water temperatures above 35 °C [27]. *A. caroliniana* can withstand a pH range between 3.5 and 10 and needs a relative humidity between 85–90% [27]. As the climate conditions during the first trial were unusually hot, with day temperatures between 28 and 35 °C, the water temperature in the system was between 29 °C ± 3 °C and pH 7.3 ± 0.1. Hence, this species was replaced in the second trial by the free-floating *Eichhornia crassipes*, which has been used previously for the effluent treatment of aquacultural wastewater, especially Nile tilapia [28]. The macrophytes in the other compartments flourished and thrived. Cyanobacteria, which were spilling over from the bloom in the hatchery water, did not thrive in the Green Liver System, probably as the macrophytes removed excess nutrients such as N and Ps and due to the inadequate conditions for cyanobacterial growth, such as shading from surface floating macrophytes.

### 2.2. Second Trial and Final Layout

*E. crassipes*, which replaced *A. caroliniana* in the first compartment for the second trial, is also a free-floating plant and thus with a similar capability for providing shade and thus cooling the water. The macrophyte is an invasive species and thus using it for phytoremediation serves as a benefit. *E. crassipes* has the enormous advantage of changing water parameters, most notably reducing the water temperature [29]. According to Greco and de Freitas [30], this macrophyte grows best at high temperatures, as experienced in Brazil. *E. crassipes* has previously been tested for the phytoremediation of urban wastewater [31], cyanide [32], and mercury [33] and has been assessed as useful and appropriate for such purposes. However, using *E. crassipes* in a Green Liver System can also have adverse effects, as this plant might reduce the oxygen level in the surface region of the water column and consequently provide the ideal environment for the development of mosquitos and snails, acting as vectors for diseases [34]. The nutrient uptake from water by *E. crassipes* is also controversial because plants seem to be able to recycle the nutrients in decaying leaves, which are still attached to the mother plant [35,36,37].

As the *E. crassipes* plants started to flower, fishnets were successfully installed close to the water surface to keep them in their compartments and to avoid spreading into the other compartments due to wind-triggered movement.

Since the wastewater comes from hatchery ponds, juvenile tilapia were flushed into the system. As they started growing and feeding on the macrophytes, the biomass within the system significantly reduced. Hence, compartments 2 to 5 had to be restocked with *Egeria densa*. To avoid the infiltration of fish via the hatchery pond systems, fish barriers were installed between the ponds and the inflow of the Green Liver System. Furthermore, a small overflow and a fish escape channel were built to avoid dead fish accumulating in front of the barrier and to allow fish to escape directly into the reservoir. To reduce the amount of tilapia already growing in the Green Liver System, a top-down approach was chosen and some predator fish were introduced into the system, specifically 30 Piranhas (*Pygocentrus nattereri*) and 10 Tucunare (common name is butterfly peacock bass; *Cichla ocellaris*). All the aforementioned preventative measures were successful in keeping each of the plants in their respective compartments and eliminating fish flowing down from the fish farm.

For Green Liver Systems, plant species with a high growth rate and the ability to produce high biomass are more efficient in the treatment of aquacultural wastewater. The *E. crassipes* and *E. densa* selected for this Green Liver System are especially capable of excessive growth. Green Liver Systems, as with most artificial systems, are easy to handle, customizable for the specific needs of stakeholders, and cost-effective. The Green Liver Systems is a suitable and promising way for aquaculture farmers to clean their wastewater before discharging them into freshwater bodies or reservoirs nearby. However, more ideas have to be developed on how to proceed with the contaminated plants. In general, when plants are saturated (up-take threshold reached), they have to be harvested to prevent die-off, which is accompanied by the possible re-release of the contaminants and their metabolites. Hence, the possibility to use the harvested plant material as fertilizer or animal feed has to be excluded.

### 2.3. Contaminant Monitoring

In general, free-floating plants should limit bloom the formation of potentially introduced cyanobacteria in the hatchery ponds as sunlight, and thus photosynthesis would be limited. Inhibited photosynthesis, in turn, will lead to the decay of the cyanobacteria and the release of any toxins, making it easier for the macrophytes in the subsequent compartments to remediate the toxins. Hence, it was expected that the submerged macrophytes, *E. densa* and *Myriophyllum aquaticum* stocked in compartment 2 to 6, would do the primary uptake of the cyanobacterial toxins. Laboratory experiments have proven the ability of both species to take up cyanotoxins [19].

Furthermore, *E. densa* and *M. aquaticum* are well known to remove heavy metals from wastewater [38,39]. Additionally, some reports exist on the removal capacity of *M. aquaticum* [40] and *E. densa* [17] with respect to the fish antibiotic oxytetracycline, which is used routinely in fish farms to keep the fish healthy despite the high fish density in the hatchery ponds as well as in the SVHD in the reservoir.

**Figure 2:**
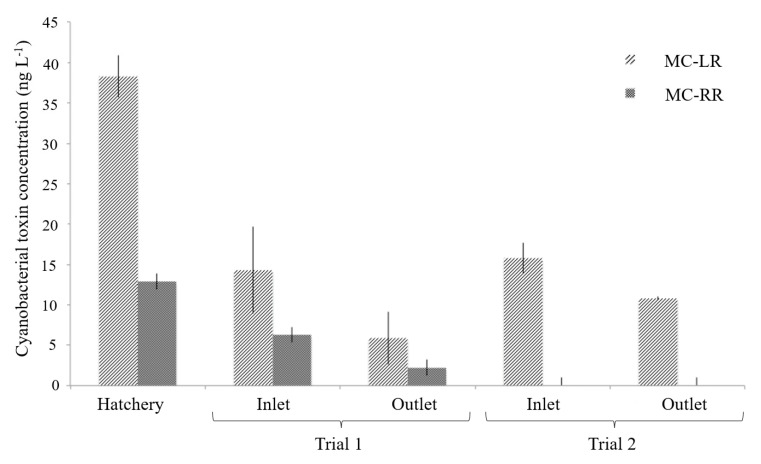
The concentration of the cyanobacterial toxins MC-LR and MC-RR in the hatchery and at the Green Liver System at the inlet (wastewater) and outlet (remediated water) during the first and second trials. Data present the average cyanobacterial toxin concentration (*n* = 7) ± standard deviation.

The first sampling results showed that the cyanotoxin burden in the hatchery water was 38.3 ± 2.6 ng·L^−1^ for MC-LR and 12.9 ± 7.3 ng·L^−1^ MC-RR. These concentrations were reduced by 58% for MC-LR and 66% for MC-RR in the first trail and by 32% MC-LR and 100% MC-RR in the second trial (Figure 2). MC-RR is ten times more toxic than MC-LR [41], making the results from the second trial more significant as 100% of the MC-RR is removed.

According to these analytical measurements of the cyanobacterial toxins, the use of a Green Liver System is an adequate and promising solution to remove contaminants from aquaculture wastewater before it is released into freshwater reservoirs. With the present system, it has been evidenced that it is an effective and low-cost way to treat fish farm wastewater. By choosing different plant species in a Green Liver System, the efficiency in exactly removing the targeted pollutants is very high. The utilization of the system in Brazil seems very practical due to the warm climate in this region; the year-round growth of the macrophytes guarantee the efficiency of the treatment [42].

In general, promising candidates from the group of macrophytes or other photosynthetic active aquatic species (ferns, moss, macroalgae) are tested in laboratory systems before being stocked in the outdoor system. This will ensure that the performance of a single plant species is known before use. In this sense, macrophytes represent the tools for the remediation of according contaminants. The selection of appropriate species has to be customized according to the contaminants in the wastewater and the requirements for the water quality to be achieved. The most crucial factor for a Green Liver System working in a semiarid and arid region seems to be the water/wastewater inflow. To ensure the adequate treatment of the wastewater, the inflow has to be relatively constant and should never be paused. Therefore, the Green Liver System can be expected to function well and sustain its performance as long as the hydrology and the growth of the macrophytes are maintained and detritus is removed from time to time.

The economic outlook is an essential factor for the sustainable cleaning-up of aquacultural wastewater as most Brazilian farmers are not able to invest vast sums of money for this issue. The required capital investment should be as small as possible, but these costs should consider the overall design and required size of the system, the construction and installation cost, the operation and maintenance and the sampling and analysis to check the performance of the system.

## 3. Conclusions

The constructed large-scale Green Liver System was not completely able to remove the MC congeners tested but could substantially diminish the MC contaminant concentrations resulting from aquacultural farms, ecologically and economically, as well as eliminate visible cyanobacterial blooms resulting from the land-based hatchery ponds before entering the Itaparica reservoir. This nature-based treatment is a first step in the right directions as untreated waste is not directly released into the environment. Other emerging contaminants in the wastewater, such as antibiotics (oxytetracycline), hormones (methyl-testosterone) and other cyanobacterial toxins will be evaluated in the future.

## 4. Materials and Methods

### 4.1. Customized Planning

**Figure A1:**
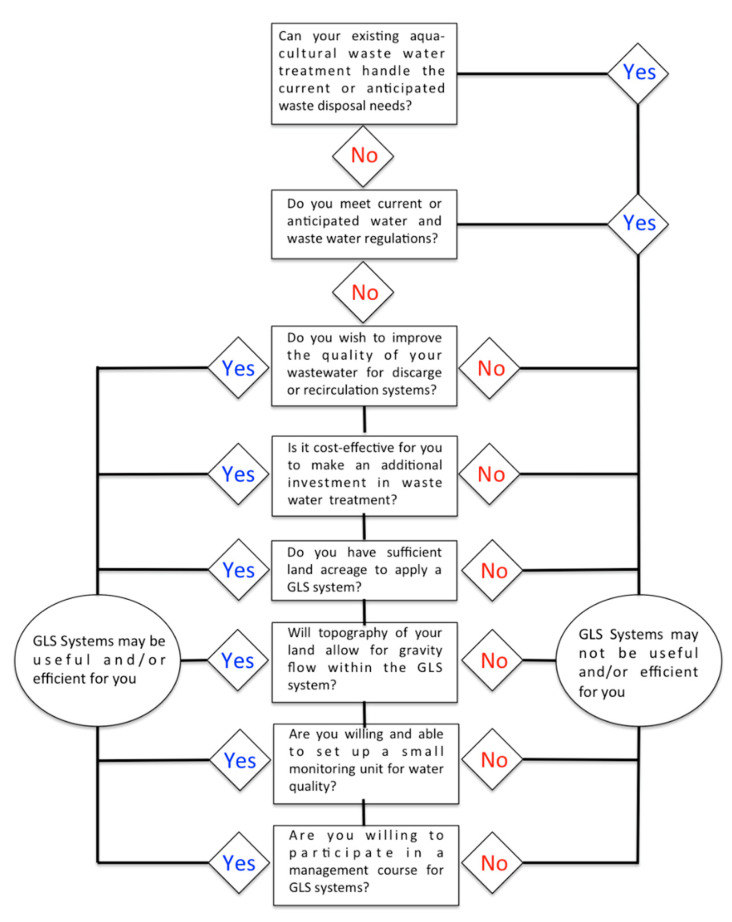
Questionnaire to figure out the needs of the stakeholder and the suitability of a Green Liver System (GLS) for their needs.

A questionnaire was developed to determine whether a Green Liver System was suitable to fit the needs of according stakeholders (Figure A1). The questionnaire excluded specific contaminant groups since they were discussed at a later stage during the customized development of a Green Liver System^®^. In this questionnaire, the more fundamental questions were asked in a simple YES or NO manner. After the first visit to COOPVALE in 2012, the area for the Green Liver System construction was defined. Since the system had to be as cost-effective as possible, no electrical pumps were used to control the water flow. Therefore, the system was planned below the hatchery pond system of the tilapia company using the natural declination to transport the wastewater from the ponds to the Green Liver System.

**Figure 3:**
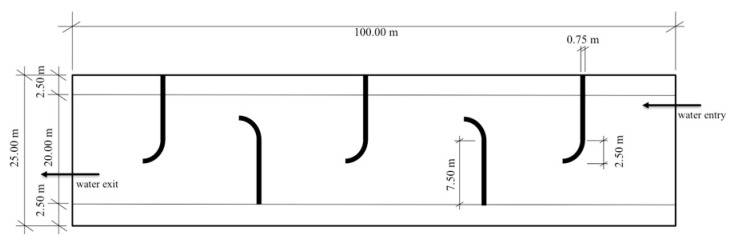
Customized construction plan for the Green Liver System on the premises of COOPVALE (Itacuruba, Brazil).

The system had a final size of 100 m × 25 m × 2 m, summating to a total volume of 5000 m^3^ (5 mL) (Figure 3). The system was divided into six compartments by curved brick stone barriers to control the water flow. Each of these barriers was 0.75 m wide to allow easy access for water sampling and management measures. The barriers, in total 15 m long, were constructed using commercially available bricks and water repellent cement. The tips of the barriers (2.5 m) were curved to minimize the accumulation of debris within the system, which further reduces the need for regular maintenance work while reducing the water flow velocity. To seal the system base, commercially available clay mineral was used at a thickness of 0.15 m. Regulated discharge from the hatchery ponds was realized through a 25 m long-channel using the natural decline from the land-based hatchery ponds, which are approximately 5 m higher than the Green Liver System. Water discharge was regulated at the hatchery ponds, to avoid the flooding of the Green Liver System.

### 4.2. Flow Calculations and Upscaling

Using the volumetric flow rate Equation (1), the required water flow rate into the system could be calculated based on the three-day retention time required by the macrophytes to remediate the pollutants as determined in the laboratory [9,10]:

(1) $$Q=\frac{V}{Rt}$$

where the flow rate (*Q*) in L·h^−1^ is equal to the bed volume (*V*) in L, divided by the residence time (*Rt*) in hours. Considering the system would operate 20 h a day, leaving four hours for maintenance or any other related works, in order for it to produce the desired volume in three days the flow rate needed in the system according to Equation (1) would be:

(2) $$Q=\frac{\left(5,000,000\;L\right)}{\left(20\;h\times 3\;\mathrm{days}\right)}=8\;3333\;L/h$$

### 4.3. Aquatic Macrophytes Used for the Green Liver System

The Green Liver System was stocked with aquatic plants found in the same area as the Itaparica reservoir, namely the macrophytes *Egeria densa* (syn. *Anacharis densa* (Planch.) Vict., *Elodea densa* (Planch.) Casp.), *Myriophyllum aquaticum* (Vell.) Verdc., and *Eichhornia crassipes* (Mart.) Solms-Laubach as well as the aquatic pteridophyte *Azolla caroliniana* (Wild.), which were previously found in laboratory studies to efficiently remediate cyanobacterial toxins as well other chemicals associated with aquaculture [10,17]. The macrophytes were allowed to acclimate for four weeks prior to the first hatchery wastewater being allowed in the system for remediation.

### 4.4. First Trial

For the first trial, *A. caroliniana*, *E. densa*, and *M. aquaticum* were used in the system. Compartment 1 and 2 were stocked with *A. caroliniana* covering half of the water surface to encourage further plant growth within the system. Compartments 3 and 4 were stocked with *M. aquaticum* using 1 kg·FW of plant material per m^2^, and in compartments 5 and 6, and *E. densa* was added at a density of 0.5 kg·FW of plant material per m^2^. The system was allowed to run for seven days before samples were taken for the first trial to track MC remediation.

### 4.5. Second Trial and Final Layout

For the second trial, in the first compartment, *A. caroliniana* was replaced by *E. crassipes*. In an attempt to avoid *E. crassipes* from spreading into the other compartments due to wind-triggered movement, fishnets were installed close to the water surface to retain the plants in their assigned compartments.

To avoid the infiltration of fish via the hatchery pond systems, fish barriers were installed between the ponds and the inflow of the Green Liver System. The fish traps were made of concrete and had two chambers filled with loose gravel stones with three different sizes (very fine, fine, and coarse) as filter material. Furthermore, a small overflow and fish escape channel were built to avoid dead fish accumulating in front of the barrier and to allow fish to escape directly into the reservoir. To reduce the amount of tilapia already growing in the Green Liver System, a top-down approach was chosen and some predator fish were introduced into the system, i.e., 30 Piranhas (*Pygocentrus nattereri*) and 10 Tucunare, more commonly known as butterfly peacock bass (*Cichla ocellaris*). The newly modified system and macrophytes were again allowed to acclimate for three weeks before the hatchery water was introduced into the system, and samples for MC congener monitoring were taken after seven days. For the final working system, macrophytes that showed any signs of chlorosis of necrosis were replaced immediately to avoid the re-release of pollutants back into the water.

### 4.6. Microcystin Congener Monitoring

Water samples for the analysis of the MC congeners MC-LR and MC-RR were taken (*n* = 7) at the fish hatchery, the Green Liver System inlet and outlet, respectively, during the first and second trials. The determination and quantification of the MC congeners, MC-LR and -RR, were performed by liquid chromatography–tandem mass spectrometry (LC–MS/MS) on an Alliance 2695 UHPLC coupled to a Micromass Quattro Micro^TM^ (Waters Corp., Herts, UK). The matrix separation was achieved on a Kinetex^TM^ C18 reverse-phase column (2.1 × 50 mm, 2.6 µm pore size, Phenomenex, Torrance, CA, USA). Milli-Q water containing 0.1% trifluoroacetic acid (TFA) and 5% acetonitrile (ACN) served as mobile phase A and ACN containing 0.1% TFA served as mobile phase B. The flow rate was maintained at 0.2 mL·min^−1^ and an injection volume of 20 µL per sample was used. The separation of the congeners was achieved using a linear gradient with the mobile phases in the following program: 0 min 65% A; 3.75–7 min 35% A and 7.8–12 min 100% A. The column oven temperature was 40 °C. Elution peaks for the MC congeners were observed at 7.1 min for MC-RR, and 7.44 min for MC-LR. The parent compound and its fragment ions, respectively, were scanned at the following mass-to-charge ratio (*m*/*z*): MC-LR 995.5 → 135.1 and MC-RR 519.9 → 135.3. ESI+ conditions for all MCs were set as follows: capillary voltage of 3 kV, source temperature of 120 °C, desolvation temperature of 500 °C and a cone gas flow rate of 100 L·h^−1^. For MC-LR, the collision energy was 65, cone voltage was 60 V, and for MC-RR the collision energy was 35, and the cone voltage was 20 V. Desolvation gas flow rate was 1000 L·h^−1^ [43]. Calibrations were linear (R^2^ = 0.999) between 5 and 500 µg L^−1^. The limit of detection (LOD) was set at 1 µg·L^−1^ (signal to noise S/N > 3) and limit of quantification at 5 µg·L^−1^ (S/N > 5) for both congeners.
